# Skeletal Muscle Mitochondrial Dysfunction in Chronic Obstructive Pulmonary Disease: Underlying Mechanisms and Physical Therapy Perspectives

**DOI:** 10.14336/AD.2022.0603

**Published:** 2023-02-01

**Authors:** Yingqi Wang, Peijun Li, Yuanyuan Cao, Chanjing Liu, Jie Wang, Weibing Wu

**Affiliations:** ^1^Department of Sports Rehabilitation, Shanghai University of Sport, Shanghai, China.; ^2^School of Physical Education and Sport Training, Shanghai University of Sport, Shanghai, China.

**Keywords:** chronic obstructive pulmonary disease, mitochondrial dysfunction, skeletal muscle dysfunction, exercise, mechanisms

## Abstract

Skeletal muscle dysfunction (SMD) is a prevalent extrapulmonary complication and a significant independent prognostic factor in patients with chronic obstructive pulmonary disease (COPD). Mitochondrial dysfunction is one of the core factors that damage structure and function in COPD skeletal muscle and is closely related to smoke exposure, hypoxia, and insufficient physical activity. The currently known phenotypes of mitochondrial dysfunction are reduced mitochondrial content and biogenesis, impaired activity of mitochondrial respiratory chain complexes, and increased mitochondrial reactive oxygen species production. Significant progress has been made in research on physical therapy (PT), which has broad prospects for treating the abovementioned potential mitochondrial-function changes in COPD skeletal muscle. In terms of specific types of PT, exercise therapy can directly act on mitochondria and improve COPD SMD by increasing mitochondrial density, regulating mitochondrial biogenesis, upregulating mitochondrial respiratory function, and reducing oxidative stress. However, improvements in mitochondrial-dysfunction phenotype in COPD skeletal muscle due to different exercise strategies are not entirely consistent. Therefore, based on the elucidation of this phenotype, in this study, we analyzed the effect of exercise on mitochondrial dysfunction in COPD skeletal muscle and the regulatory mechanism thereof. We also provided a theoretical basis for exercise programs to rehabilitate this condition.

Chronic obstructive pulmonary disease (COPD) is a commonly recognized respiratory disease characterized by persistent and progressive expiratory-airflow limitation [1]. However, studies show that in addition to respiratory symptoms, patients with COPD often present with at least one extrapulmonary complication such as osteoporosis, skeletal muscle dysfunction (SMD), and cognitive impairment, resulting in malignant development of COPD [2]. As one of the systemic effects of COPD, patients with SMD have poor muscle strength, low muscle mass/quantity, and limited physical activity [3]. A meta-analysis reported that the clinical prevalence of skeletal-muscle damage in COPD is 21.6% in general and 53.8-66.7% in nursing homes. SMD is closely related to degree of airflow limitation, dyspnea score, exacerbation frequency, and mortality in these patients [4-8].

Because COPD SMD has multiple adverse consequences, exploring the underlying mechanism of SMD can help improve patients’ survival status. Mitochondrial-phenotype changes are a crucial link in skeletal muscle’s structural and functional impairment in COPD [9]. Structurally, the decrease in number of mitochondria per unit area of skeletal muscle in COPD patients changes the proportion of skeletal-muscle fiber types from oxygen-rich type I fibers to type II fibers, which are dominated by anaerobic metabolism. This can cause muscle atrophy in COPD [10, 11]. Functionally, cigarette smoke and its secondary effects can increase oxidative stress (OS) in skeletal-muscle mitochondria and reduce muscle contractility [12]. OS is negatively correlated with COPD skeletal-muscle endurance [13]. The vastus lateralis of patients with COPD shows decreased mitochondrial-oxidase activity and significantly decreased oxidative capacity, which are associated with decreased muscle exercise capacity and poor endurance [14, 15]. Moreover, insufficient mitochondrial adenosine triphosphate (ATP) production and increased ATP consumption could be the main causes of the decline in skeletal-muscle function. Studies show that smoking can reduce the activity of the mitochondrial respiratory chain (MRC), hinder the production of mitochondrial ATP, and possibly increase muscle fatigue [16]. The greater levels of ATP consumed by muscle contraction can then lead to poor exercise capacity in COPD patients [17].

In recent years, the role of physical therapy (PT) in the management of COPD has become prominent [18]. As an essential part of COPD pulmonary rehabilitation, exercise positively affects COPD skeletal-muscle strength, mass, and physical activity [19, 20]. Mitochondria are the source of energy for life activities. The improvement of SMD in COPD by exercise might be related to exercise’s regulation of mitochondria. Studies find that endurance exercise can enhance the mitochondrial-antioxidant defense system, reduce the toxic effects of OS on skeletal muscle, and prevent muscle weakness [21]. However, the specific mechanism by which exercise improves mitochondrial dysfunction in COPD skeletal muscle remains unclear. Therefore, this paper illustrates mitochondrial dysfunction of COPD skeletal muscle and its relationship with skeletal-muscle damage, analyzes the effect of exercise on mitochondrial dysfunction in COPD and the possible underlying regulatory mechanism, and provides a theoretical basis and practical exercise rehabilitation program for the improvement of COPD SMD.

## Mitochondrial dysfunction in COPD skeletal muscle

Mitochondria are double-membrane organelles that undertake the tasks of cell material synthesis, energy conversion, and information exchange. They have three primary functions: ATP generation, Ca^2+^ storage and release, and generation and scavenging of oxidative substances [22]. When mitochondrial function is unbalanced in COPD, skeletal-muscle structure, and function change accordingly, with sequelae such as skeletal-muscle atrophy and decreased muscle endurance [14]. Even under the same physical activity, patients with COPD SMD show remarkable changes in mitochondrial-unit density and biogenesis, impaired mitochondrial respiration, and increased mitochondrial OS [23].

## Reduced density and altered biogenesis of mitochondria

In the skeletal muscle of COPD patients, decreases in number of mitochondria per unit area and in biogenesis are frequently observed [24]. Reduced activity of citrate synthase, a biomarker of mitochondrial density, indicates insufficient mitochondrial oxidative capacity and may contribute to reduced quadriceps endurance [25]. Studies have found that the oxidative capacity of upper- and lower-extremity muscles is reduced in patients with moderate to severe COPD [26]. The activity of mitochondrial citrate synthase is reduced by up to 37% when skeletal-muscle exercise capacity and muscle mass are lower in COPD patients, showing that the decrease in number of mitochondria per unit area is closely related to exercise intolerance [11].

Changes in mitochondrial content and apparent changes in muscle fiber types are the main manifestations of mitochondrial adaptation to external stimuli. The entire adaptation process is called mitochondrial biogenesis [27]. Peroxisome proliferator-activated receptors (PPARs) and PPAR-γ co-activator 1-alpha (PGC-1α), which are involved in mitochondrial-energy conversion and biogenesis, are essential regulators of signaling pathways related to skeletal-muscle structure and function [28]. Studies have found that knockout of PGC-1α can lead to decreased endurance and muscle fiber damage in mice [29]. Meanwhile, PGC-1α overexpression can reduce expression levels of muscle really interesting new gene-finger 1 (MuRF-1) and atrogin-1 messenger ribonucleic acid (mRNA) in the ubiquitin/proteasome system (UPS) to relieve muscle atrophy in mice [30, 31]. The mitochondrial transcription factor A (TFAM) is also involved in the process of mitochondrial biogenesis and is a vital part of the regulatory mechanism of PGC-1α [32]. Compared with healthy subjects, patients with COPD have decreased skeletal-muscle PPARδ content, TFAM expression levels, and PGC-1α mRNA expression. PPARα mRNA expression further decreases as severity of COPD increases [33, 34]. Studies suggest that COPD can lead to abnormal expression of mitochondrion-related signaling molecules, decreased mitochondrial content, and biogenetic disorders, resulting in COPD SMD.

## Impaired mitochondrial respiration

Mitochondrial respiration is the most efficient energy metabolism mechanism in skeletal muscle. It depends on the MRC, which consists of complexes I-IV and is located on the inner mitochondrial membrane. When electrons transfer through the MRC, the MRC enzyme complex V (*i.e.*, ATP synthase) utilizes the released energy to catalyze the oxidative phosphorylation of adenosine diphosphate (ADP) and produce ATP [35, 36]. However, impaired activity of MRC complexes in COPD skeletal muscle often decreases mitochondrial respiratory function (MRF), affecting muscle structure and function [37]. COPD patients with different body mass indices (BMIs) have inconsistent manifestations of skeletal-muscle mitochondrial respiratory dysfunction. Studies show that the MRC and oxidative-phosphorylation indicators are significantly decreased in normal- and low-weight patients with COPD [38]. Compared with normal-BMI COPD patients, those with low BMI have significant MRC dysfunction in peripheral muscles, which might lead to decreased muscle endurance [39]. However, mitochondrial density does not change in normal-weight patients [38]. This phenomenon suggests that the decline in MRF due to impaired MRC complex activity in COPD skeletal muscle precedes changes in mitochondrial density and ultimately leads to SMD. In addition, Zhang et al. found that the expression level of PPARα mRNA is correlated with mitochondrial respiratory dysfunction and decreased mitochondrial content in muscle cross-sectional areas [38]. The PPAR signaling pathway might therefore regulate MRF.

## Increased production of mitochondrial reactive oxygen species

Reactive oxygen species (ROS) are involved in various types of signal transduction in cells. Low levels of ROS can play a protective role as an essential cellular signal [40]. However, as levels of ROS increase, studies observe imbalance between intracellular oxidants and antioxidants, disrupted redox signaling pathways, and activated OS. These changes can selectively target the generation of ATP and the storage and release of Ca_2_^+^, resulting in molecular-structure and -function damage in mitochondrial proteins, deoxyribonucleic acid (DNA), and lipids [41]. Mitochondrial damage generates more ROS, forming a vicious cycle that eventually activates muscle atrophy-related pathways and apoptosis, further aggravating SMD [42-44].

OS in COPD skeletal muscle comes from exogenous and endogenous pathways. Exogenous OS comes from harmful substances, such as free radicals and carbon monoxide in smoke. Endogenous OS is derived from the self-defense response of inflammatory cells and proliferation of ROS [45]. Mitochondria are involved in the production of most ROS and are an important hub of OS [46]. Current studies suggest that mitochondrial complexes I and III are the main sites of ROS production [47]. Mitochondrial complex III has been confirmed as the primary site of increased ROS levels in the skeletal muscle of COPD patients, which can lead to increased muscle OS [48]. In addition, mitochondrial-electron leakage precisely regulated by uncoupling proteins (UCPs) affects ROS production. Five different kinds of UCPs have been found so far. Studies indicate that an appropriate amount of UCP3 can reduce mitochondrial membrane potential (MMP; Δψ) to inhibit ROS release, preventing excessive OS in the body and minimizing damage by lipid peroxides to mitochondria [22]. However, levels of UCP3 in the skeletal muscle of COPD patients are reduced, suggesting that the accumulation of lipid peroxides in skeletal muscle might cause excessive mitochondrial OS and insufficient exercise capacity in these patients [49, 50].

## Influencing factors of mitochondrial dysfunction in COPD skeletal muscle

The mitochondrial dysfunction of COPD skeletal muscle is caused by a combination of factors, predominantly related to smoke exposure, hypoxia, and insufficient physical activity.

### Smoke exposure

Harmful exposure factors such as smoking are the primary causes of COPD. Smoking severely damages the normal physiological structure of the lungs, causes breathing difficulties, and induces a series of toxicological damages to skeletal muscles via local and systemic effects [51]. Studies find that even mild smoke exposure can significantly reduce quadriceps strength in patients with COPD. SMD precedes respiratory damage caused by smoking, which suggests that the toxic damage caused by smoke can directly act on skeletal muscle[46]. The underlying mechanism might be that harmful substances in cigarettes promote mitochondrial dysfunction. Alonso et al. showed that skeletal-muscle mitochondrial complex IV is inhibited, and MRF is impaired after smoking in healthy subjects [16]. In addition, many studies confirm that smoke exposure increases mitochondrial OS and induces phenotypic changes in skeletal muscle, which can impair mitochondrial-oxygen transport and utilization and ultimately cause muscle weakness [52, 53].

### Hypoxia

Oxygen is vital to maintenance of normal skeletal-muscle mitochondrial function. Hypoxia is a common predisposing factor for SMD in patients with COPD, mediating damage to mitochondrial structure and function. Sauleda et al. found that in hypoxemia, the activity of complex IV, as a terminal enzyme of the MRC, is altered in COPD patients and is inversely related to arterial oxygen partial pressure (PaO_2_) [54]. This finding suggests that hypoxia might upregulate MRC enzymes, resulting in impaired MRC activity [55]. Hypoxia can also increase mitochondrial-OS indicators in skeletal muscle and impair oxidative-phosphorylation capability. The antioxidant vitamin E can downregulate mitochondrial OS and prevent further aggravation of mitochondrial dysfunction [56]. In addition to mitochondrial abnormalities, prolonged exposure to hypoxia adversely affects mitochondrial metabolism in patients with COPD, resulting in decreased mitochondrial-enzyme activity and reduced mitochondrial content [57].

### Insufficient physical activity

Physical inactivity is prevalent in patients with COPD and pronounced during exacerbations of the disease [58]. Physical activity in COPD decreases significantly with age and seriously affects patients’ quality of life (QoL) and prognoses [59]. Current studies find that insufficient physical activity greatly affects mitochondrion-related energy metabolism, causing changes in mitochondrial morphological structure, decreased mitochondrial number, impaired MRF, and increased OS [52, 60, 61]. Prolonged sitting in the elderly can significantly reduce PGC-1α and the activity of MRC enzymes, which suggests that insufficient physical activity might affect mitochondrial biogenesis and MRF [62]. A study found that sedentary mice have increased mitochondrial uncoupling and oxidative damage that contribute to mitochondrial dysfunction in skeletal muscle compared with aged mice that undergo exercise [63]. However, whether smoking or sedentary behavior is the leading cause of mitochondrial dysfunction in COPD skeletal muscle is unclear. Based on currently available evidence, these results might suggest long-term gradual changes in mitochondrial OS due to smoking and that skeletal-muscle metabolic abnormalities in COPD are mainly attributable to insufficient physical activity [52]. This area needs additional research.

## Effects of exercise on mitochondrial dysfunction in COPD skeletal muscle

Exercise, a core PT for SMD in COPD patients, can ameliorate mitochondrial dysfunction by improving mitochondrial phenotype and redox status.

Different exercise strategies offer different degrees of mitochondrial regulation. One study found that eccentric exercise in patients with COPD can significantly improve muscle strength and mass, but no considerable change in mitochondrial function was observed. Meanwhile, concentric exercise can significantly change mitochondrial biogenesis and MRF [64]. A possible explanation is that eccentric and concentric exercises have different cardiorespiratory loads and metabolic oxygen demands. With the same degree of exertion, eccentric exercise can change the body’s metabolic substrates, promote oxygen release in the circulatory system, and lower metabolic cost and cardiopulmonary oxygen consumption [65-67]. Conversely, concentric exercise requires greater oxygen supply and higher cardiopulmonary function, which is beneficial to improving mitochondrial ATP production and MRF [64, 68].

Studies have also found that despite no significant change in skeletal-muscle mitochondrial density after exercise in patients with COPD, single-joint exercise in these patients can elevate skeletal-muscle maximal oxygen uptake and muscle oxygen supply more than whole-body exercise [69]. This result is also related to limitation of cardiorespiratory function and adequacy of oxygen supply and utilization. COPD patients have 43% higher pulmonary-oxygen consumption costs and significantly lower power output during whole-body exercise, resulting in a particular limitation of the local muscle oxygen supply and a mismatch between oxygen availability and mitochondrial respiration rate. This limitation and mismatch further exacerbate the cost of ATP consumption and higher energy supply during muscle contraction [70]. In contrast, single-joint exercise is less limited by cardiorespiratory fitness. Despite reduced skeletal-muscle blood supply and diffusion to mitochondria in the initial stage of COPD, studies have demonstrated that 6 weeks of single-joint exercise restores vastus lateralis MRF and peak power to normal levels in COPD patients [71, 72]. Therefore, both single-joint and whole-body exercises can increase skeletal-muscle mitochondrial adaptability, but single-joint exercise has more robust mitochondrial adaptability than whole-body exercise. The abovementioned studies indicate that the degree of oxygen supply and utilization in the circulatory system and muscle tissue during exercise are important reasons for the differences in mitochondrial regulation between exercise strategies in COPD patients.

Different exercise styles have similar effects on mitochondrial improvement. Endurance exercise has long been the preferred method for pulmonary rehabilitation and has the characteristics of repetition, duration, and low intensity. Maltais et al. demonstrated that endurance exercise, specifically 12 weeks of cycling training, reduces exercise-induced lactic acidosis and improves the oxidative capacity of skeletal muscle in patients with moderate to severe COPD [73]. In addition, 8 weeks of endurance training can increase citrate synthase activity in the skeletal muscle of COPD patients, increase mitochondrial density, and improve the body’s oxidative capacity [74]. Compared with endurance exercise, resistance exercise is a high-intensity, low-frequency form of anaerobic training that targets local muscles. Ryrsø et al. found increased levels of skeletal-muscle antioxidant superoxide dismutase 2 (SOD2), which upregulates muscle antioxidant capacity, in patients with moderate to severe COPD after 8 weeks of endurance and resistance training. No significant difference was seen between the effects of both types of exercise [75]. Interestingly, another study confirmed that combined endurance/resistance exercise in patients with COPD remarkably improves exercise capacity, skeletal-muscle mito-chondrial function, and muscle fiber cross-sectional area compared with the single-exercise method [76]. In conclusion, there appears to be no difference between endurance and resistance exercise in regulating mitochondrial oxidative and antioxidant capacity in the skeletal muscle of COPD patients; both types of exercise can balance mitochondrial redox status. The effect of the combined exercise was better than that of a single exercise, which might have been due to the additive effect of both types.

The effects of different exercise intensities on mitochondrial dysfunction are also similar. High-intensity interval training is effective in PT for patients with COPD [77]. One study in which participants underwent regular exercise training for 10 weeks confirmed that both high-intensity interval exercise and moderate-intensity constant load training could induce mitochondrial-function changes and increase peripheral muscle cross-sectional areas in advanced-COPD patients. High-intensity interval exercise has certain advantages in reducing training symptoms [78]. In contrast, Van et al. found that a single session of maximal and submaximal exercise increased levels of inflammation and ROS production and reduced antioxidant activity in COPD patients with muscle wasting compared with healthy subjects and COPD patients with normal skeletal muscle [79]. The inconsistency of these studies might be related to the amount of single maximal exercise and the state of the skeletal muscle itself in COPD patients. The pathological state of muscle wasting might affect how exercise regulates inflammatory responses and OS. In addition, low-intensity exercise can improve mito-chondrial oxidative phosphorylation in COPD patients, thereby increasing respiratory oxidative control [80]. This comparison of the potential effects of different exercise intensities indicates that high-intensity interval training might be more suitable for rehabilitation of COPD SMD due to regular changes in exercise intensity, fluctuations in oxygen uptake and utilization, and mild training symptoms.

In conclusion, exercise’s ability to improve mitochondrial dysfunction in COPD skeletal muscle cannot be ignored. The current evidence shows that the effects of different exercises on mitochondrial dysfunction in skeletal muscle in COPD patients are not entirely consistent. Both concentric and single-joint exercise can better promote mitochondrial respiratory improvement. Eccentric exercise maximizes skeletal-muscle function, both endurance exercise and resistance exercise can better restore the mitochondrial redox state of COPD skeletal muscle, and high-intensity interval training may be better at improving mitochondrial dysfunction in these patients.

Taking the above into account, exercise rehabilitation programs for COPD patients should be formulated based on patient cardiopulmonary function, disease severity, and maximization of benefits. For COPD patients with poor exercise tolerance and impaired cardiorespiratory function who are in the initial stage of enhanced physical activity, we recommend eccentric exercise and single-joint exercise that exchange low cardiorespiratory load and low metabolic cost for muscle strength and quality. COPD patients with relatively good exercise ability can choose concentric, endurance, and resistance exercises that enhance mitochondrial function better. Exercise intensity should be based on the patient’s exercise tolerance. No studies have yet definitively identified the best exercise for COPD patients. As discussed above, the optimal exercise strategy combines different exercise modalities, enhancing patient muscle function and potential effectors, for example, the combination of concentric and eccentric exercises or of endurance and resistance exercises. The relevant literature on the effect of exercise on mitochondrial dysfunction in skeletal muscle is shown in Table 1.

## Mechanisms by which exercise improves mitochondrial dysfunction in skeletal muscle

### Exercise improves mitochondrial density and biogenesis

Normal mitochondrial density and biogenesis are essential for energy metabolism in skeletal muscle. Long-term regular exercise can increase mitochondrial number and biogenesis, resulting in good adaptation of muscle cells [81]. This result is probably due to increased skeletal-muscle blood flow and oxygen transport; regular long-term exercise can meet the oxygen uptake requirements of muscle tissue, thereby promoting mitochondrial adaptive changes. Studies find that compared with double-leg training, exercise of a single limb in stable patients with COPD can improve maximum oxygen uptake, suggesting that local training is more effective than full-body training in improving mitochondrial density and biogenesis [82].

**Table 1:** Effect of exercise on mitochondrial dysfunction in COPD skeletal muscle.

Study	Group	N	Exercise style	Exercise intensity	Exercise duration	Mitochondrial dysfunction indicators	Skeletal muscle function indicators
MacMillan et al., 2017^[64]^	EET COPD group CET COPD group	8/7	recumbent cycle ergometer	60-80% peak workrate	10 weeks, 3 days/w, 30 min/day	EET: no effect CET: PGC-1α↑, PGC-1αmRNA↑, Complex I↑, Complex II↑, State3 (Complex 1 driven)↑, State 3 (Complex I and II driven)↑, Complex IV-driven respiration↑	EET: isometric peak strength↑, Isometric peak strength normalized↑, Total isokinetic work↑ CET:no effect
Richardson et al.,2004^[69]^	COPD group Control group	6/6	maximal bicycle exercise and single leg knee-extensor exercise	Bicycle:60-80 r/min Maximum knee extension	8-12 min	O_2_ delivery and uptake↑, stable oxygen content, partial pressure of oxygen↑	type I fibers↑, VO2max↑
Maltais et al., 1996^[73]^	COPD group	11	cycle ergometer	80%VO_2_max	12 weeks, 3 days/w, 30 min/day	CS↑, HADH↑	VO_2_max↑, The lactate threshold↑ V_E_↓, Arterial lactic acid concentration↓
Gosker et al., 2006^[74]^	COPD group Control group	13/7	resistance and endurance training	Activity of Daily Living and muscle performance	8 weeks, 5 days/w, 100 min/d	CS↑, UCP3↑, HADH↑ lipid peroxidation level	VO_2_peak↑, peak workload↑
Ryrsø et al., 2018^[75]^	COPD group Control group	30/8	resistance or endurance training	Level 14-15 on the Borg scale of perceived exertion	8 weeks, 3 days/w, 35 min	SOD2↑, NOX	VO_2_peak↑, Wmax↑, 6MWD↑
Brønstad et al.,2012^[71]^	COPD group Control group	7/5	single leg knee-extensor exercise	90% of peak load	6 weeks, 3 days/w	CS↑, maximal mitochondrial respiration↑	VO2peak↑
McKeough et al.,2006^[76]^	COPD group Control group	10/10	Leg cycling+ walking+strength training	80% peak workload +80% walking Speed+80% of 1RM	8 weeks, 2 days/w, 70 min	PCr↑, PCr *t*_1/2_ predicted↑, ADP	6MWD↑, peak workload↑, CSA↑, MVC↑
Van et al., 2006^[79]^	Non-muscle-wasted COPD group Muscle-wasted COPD group Control group	10/10/10	maximal incremental bicycle until exhaustion; submaximal bicycle	5-20 W/min increased until exhaustion; 50% of Wmax	Submaximal bicycle 30 min at least	protein oxidation↑, ROS↑, GSSG/GSH↑, lipid peroxidation (muscle-wasted group) plasma antioxidant capacity↓, GSH↓	VO_2_↓, V_E_↓, Workload↓ MVC
VOGIATZIS et al., 2005^[78]^	IE COPD group CLE COPD group	10/9	electromagnetical braked cycle ergometers	IE: 124±15% CLE: 75±5% Wpeak	10 weeks, 3 days/w, 45/30 min	CS activity↑, PFK activity	CSA↑, Wpeak↑, lactate threshold↑, capillary-to-fiber ratio↑
GUZUN et al., 2012^[80]^	COPD group Control group	8/8	Supervised home exercise training	progressively increased from 50 to 80% of Pmax	12 weeks, 3 days/w, 45 min	metabolic power↑, maximal rate of ADP-stimulated respiration, JO2Max, app. KmADP↓	maximal cycling mechanical power↑, maximal tidal volume↑, CSA

↑: The value of experimental group was significantly higher than that of control group. ↓: The value of experimental group was significantly lower than that of control group. COPD, chronic obstructive pulmonary disease; EET, Eccentric ergometer training; CET, concentric ergometer training; VO_2_max, maximal oxygen consumption; CS, citrate synthase; HADH, 3-hydroxyacyl-CoA dehydrogenase; V_E_, minute ventilation volume; VO_2_peak, peak oxygen consumption; UCP3, uncoupling protein-3; Wmax, maximal workload; 6MWD, 6-minute walking distance; 1RM, one repetition maximum; PCr, phosphocreatine concentration; CSA, cross-sectional area; MVC, maximum voluntary contraction; PCr *t*_1/2_, phosphocreatine recovery half-time; ROS, reactive oxygen species; GSH, Glutathione; GSSG, oxidized glutathione; IE, interval exercise; CLE, constant-load exercise; Wpeak, peak work rate; PFK, phosphofructokinase; Pmax, maximal mechanical power; JO2Max, maximal ADP-stimulated (2 mm) respiration rates; app. KmADP, The apparent Michaelis constant for ADP.

The effect of exercise on COPD skeletal-muscle mitochondrial biogenesis is closely related to activation of PGC-1α-related signaling pathways. PGC-1α inhibits the ubiquitin proteolytic system and myostatin to reduce muscle protein degradation and is a signaling node for exercise-triggered mitochondrial density and biogenesis [83]. Among the many regulatory factors upstream of PGC-1α, adenosine monophosphate-activated protein kinase (AMPK) is an essential regulatory protein of skeletal-muscle energy metabolism during exercise and copes with energy shortage [27]. Studies show that exercise transmits signals to PGC-1α through AMPK, resulting in increased PGC-1α protein expression and mitochondrial biogenesis [84]. Other studies demonstrate that moderate-intensity exercise can increase the expression of PGC-1α in the skeletal muscle of COPD patients, further regulating mitochondrial biogenesis. In exercise below the lactate threshold, decreased mitochondrial DNA and PGC-1α mRNA overexpression can still be observed, although the degree of change is small [33]. During the entire adaptation process, activation of PGC-1α further promotes upregulation of the downstream regulator TFAM, preventing degradation of mitochondrial DNA and activating mitochondrial-protein transcription [85]. In addition, as a highly expressed transcription factor in skeletal muscle, PPARα is closely associated with mitochondrial adaptive changes, has a strong regulatory ability in skeletal-muscle energy metabolism, and affects expression of PGC-1α signaling. Therefore, exercise may improve mitochondrial density and biogenesis in COPD by activating AMPK upstream proteins and promoting PGC-1α expression. PGC-1α plays a key role through transcriptional regulation of its downstream factor TFAM.

### Exercise enhances mitochondrial respiratory function

Mitochondria are where the energy required for COPD skeletal-muscle movement is generated, and normal MRF is a necessary condition for skeletal-muscle exercise capacity. During exercise, the MRF of skeletal muscle is upregulated. ADP and mitochondrial substrate concentrations are significantly increased during exercise compared with the resting state, thereby promoting generation of ATP to continuously supply energy [86]. Studies show that improvement in exercise capacity is related to the increase in mitochondrial content and mitochondrial oxidative capacity of skeletal muscle in normal subjects [87], which might also apply to patients with COPD [39].

Numerous studies demonstrate that exercise can improve mitochondrial dysfunction in COPD skeletal muscle by upregulating MRF. After a 7-week rehabilitation process combining endurance and strength exercises, Calvert et al. found reduced blood ammonia levels and lactate accumulation and loss of adenine nucleotides in COPD patients, which increased ATP synthesis and fully supplied the body’s energy metabolism [88]. This finding might be due to inhibition of muscle anaerobic capacity caused by the enhanced mitochondrial oxidative capacity. Compared with whole-body exercise, localized exercise can similarly improve MRF. Studies show that even exercise training targeting a specific muscle group might improve MRF and patient exercise capacity [71]. However, comparative studies on differences in improvement of MRF between whole-body and local exercises are lacking, and further exploration is needed to clarify which range of exercise has the better effect.

The normal work of mitochondrial respiration depends on the MRC. Mitochondrial complex I is the largest protein complex on the inner mitochondrial membrane and is responsible for 95% of mitochondrial respiration [52]. Previous studies indicate that impaired mitochondrial respiration predominantly manifests as reduced mitochondrial complex I respiration efficiency [23]. Recent experiments in mice exposed to tobacco smoke also confirm that smoke exposure significantly impairs complex I-mediated MRF in a dose-dependent manner [89]. These results correspond to the findings of Brønstad et al. The improvement of skeletal-muscle MRF in patients with COPD by exercise is predominantly due to increased mitochondrial mass and improved mitochondrial complex I activity [71]. This finding leads us to speculate that mitochondrial complex I could be an essential target in exercise’s regulation of MRF. Exercise might improve regulation of skeletal-muscle mito-chondrial respiration by targeting mitochondrial complex I and further accelerate muscle state recovery in patients with COPD.

### Exercise reduces mitochondrial ROS generation and modulates redox status

The redox reaction is the electron transfer process between oxidants and antioxidants. As important carriers of the redox reaction, mitochondria go through a series of redox states, reflecting the level of cellular metabolism [90]. Therefore, maintaining mitochondrial redox state balance is key to body homeostasis.

Under constant stimulation of ROS production both externally and internally, the body’s antioxidant defense system plays an important role in inhibiting ROS. Antioxidant defense systems are divided into two categories: enzymatic antioxidants, such as SOD, catalase, and glutathione peroxidase (GSH-Px); and nonenzymatic antioxidants, such as vitamins C and E [91]. An experimental study showed that aerobic exercise decreases total mitochondrial superoxide/H_2_O_2_ production in skeletal muscle compared with the resting state [92]. Consistent with this finding, the rate of mitochondrial H_2_O_2_ production is slow during muscle contraction, which might be due to enhanced catalase activity [93], *i.e.*, enhanced antioxidant scavenging. Current research finds that the enhanced ROS-scavenging effect of the body’s antioxidants is related to nuclear factor erythroid 2-related factor 2 (Nrf2). When the body is stimulated by excessive ROS, the upstream protein Kelch-like epichlorohydrin (ECH)-associated protein 1 (Keap1), which regulates cellular localization and Nrf2 content, is inhibited. The bound Nrf2 is released and transferred to the nucleus, where it interacts with antioxidant response element (ARE). The combination of Nrf2 and ARE increases the activity of antioxidant enzymes, neutralizes excess ROS, and regulates redox state balance [94, 95]. Toledo et al. found downregulated oxidative expression of glutathione and upregulated total antioxidant capacity and Nrf2 expression in the skeletal muscle of mice exposed to smoke after 12 weeks of aerobic exercise [96]. These results indicate that exercise can activate the Keap1-Nrf2-ARE signaling pathway, upregulating the antioxidant capacity of skeletal muscle, and reducing mitochondrial OS, effects which increase over time.

To the best of our knowledge, exercise might enhance the mitochondrial-antioxidant defense mechanism of COPD skeletal muscle to inhibit ROS generation and lower OS by regulating mitochondrial-electron leakage through UCPs. Studies show that exercise can restore UCP3 content in the muscles of COPD patients, alter ROS generation, improve oxidative capacity, and protect mitochondria from the toxic effects of OS [74]. However, other studies suggest that patients with COPD have abnormal skeletal-muscle redox status and increased OS after exercise [97-99]. This inconsistency might be related to COPD severity, different exercise ranges (whole-body or local), and whether exercise is single-time or regular. Severe COPD or single local-exercise intervention in COPD patients is more likely to increase OS, but the specific mechanism needs further study. In conclusion, exercise enhances the mitochondrial antioxidant capacity of COPD skeletal muscle by activating the Keap1-Nrf2-ARE pathway and inhibits ROS generation by upregulating the content of UCP3, thereby dynamically balancing the redox state.

Interestingly, in recent years, more studies have focused on whether the underlying mechanisms by which different exercises affect mitochondrial metabolism are similar [100]. As demonstrated in this article, there are inconsistencies in that different exercises improve mitochondrial dysfunction in COPD skeletal muscle. Concentric, single-joint, and whole-body exercises chiefly upregulate mitochondrial density and MRF in COPD skeletal muscle. Endurance and resistance exercises mainly regulate mitochondrial respiratory metabolism and the redox state of skeletal muscle in these patients. Different exercise intensities mainly focus on mitochondrial respiratory control and OS changes. These findings reflect that the different types of exercise can stimulate muscle function. However, the underlying mechanism of improving mitochondrial dysfunction in COPD has differences in mitochondrial density and biogenesis, MRF, and mitochondrial OS. Therefore, the abovementioned research has limitations, and whether the specific mechanism differs among different types of exercise needs follow-up study.

**Figure 1.**
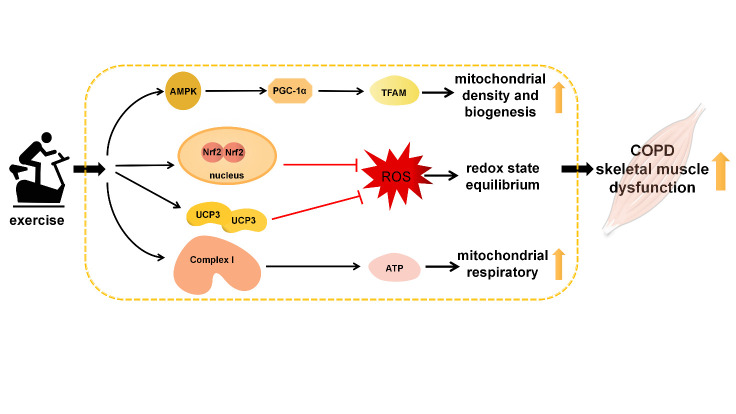
The mechanism by which exercise improves mitochondrial dysfunction in COPD skeletal muscle. Exercise might increase mitochondrial density and biogenesis, inhibit ROS production, and regulate mitochondrial respiration by: (1) activating the AMPK-PGC-1α-TFAM pathway; (2) activating the Nrf2 signaling pathway and upregulating UCP3; and (3) targeting mitochondrial complex I and increasing ATP. COPD, chronic obstructive pulmonary disease; AMPK, adenosine monophosphate-activated protein kinase; PGC-1α, peroxisome proliferator-activated receptor γ coactivator-1α; TFAM, mitochondrial transcription factor A; Nrf2, nuclear factor erythroid 2-related factor 2; UCP3, uncoupling protein 3; ROS, reactive oxygen species; and ATP, adenosine triphosphate.

## Conclusions and perspectives

The occurrence of SMD in COPD is universal, adjustable, and related to the degree of respiratory symptoms. It can easily lead to various adverse consequences such as falls and fractures. Among the many factors contributing to skeletal-muscle structure and function impairment in COPD, mitochondrial dysfunction is at the center of the pathogenic mechanism network, directly affecting muscle energy metabolism. From the perspective of mito-chondrial damage, various studies have found that it influences muscle strength and endurance, which nicely explains the internal relationships between molecular mechanisms, especially in the presence of OS. Smoke exposure, hypoxia, and insufficient physical activity are important risk factors affecting mitochondrial function. The interaction of multiple factors ultimately changes mitochondrial density and biogenesis, decreases MRC function, and increases mitochondrial ROS production. However, the underlying mechanism linking mito-chondrial dysfunction with impaired structure and function in COPD skeletal muscle is not fully understood. Understanding mitochondrial dysfunction in COPD skeletal muscle is a critical premise and challenge in exploring the COPD therapeutic principle and effect, which will help identify a precise target in future studies.

To the best of our knowledge, this article is the first to delve into the underlying mechanisms by which exercise improves mitochondrial dysfunction in COPD skeletal muscle. Notably, targeting mitochondrial dysfunction in COPD skeletal muscle through exercise training is prospective. Exercise is a non-drug treatment that effectively promotes an increase in mitochondrial density and biogenesis, improves MRF, and regulates redox status, meaning that it can reverse mitochondrial dysfunction and prevent further loss of skeletal muscle. We also recommend appropriate exercise options for COPD patients with different disease severities and exercise abilities, which can provide a reference for clinical application. Based on the existing research, the combination of different exercise strategies such as endurance and resistance exercises seem to improve various functional indicators of mitochondria, thereby restoring the optimal state of COPD skeletal muscle. However, relatively few studies focus on exercise in terms of mitochondrial phenotype and the molecular mechanism of changes in COPD skeletal muscle. No study has comprehensively compared the improvement effects of different exercise times, intensities, and forms, nor has any particular study focused simultaneously on the three aspects of mitochondrial-phenotype changes improved by exercise. Therefore, the effects of different exercise patterns, durations, and intensities on mitochondrial damage should be further studied. In future work, we will focus on mitochondria to explore the exact mechanism by which exercise improves treatment effect in COPD patients, providing new ideas for exercise rehabilitation programs.
